# A Study of the Use of Laryngeal Mask Airway (LMA) in Children and its Comparison with Endotracheal Intubation

**Published:** 2009-04

**Authors:** Shahin N Jamil, Mehtab Alam, Hammad Usmani, M M Khan

**Affiliations:** 1 Reader, Department of Anaesthesiology, J.N. Medical College, AMU, Aligarh (U.P); 2 Ex-Lecturer, Department of Anaesthesiology, J.N. Medical College, AMU, Aligarh (U.P); 3 Lecturer, Department of Anaesthesiology, J.N. Medical College, AMU, Aligarh (U.P); 4 Junior Resident., Department of Anaesthesiology, J.N. Medical College, AMU, Aligarh (U.P)

**Keywords:** Laryngeal mask airway, Endotracheal intubation, Children

## Abstract

**Summary:**

Laryngeal mask airway (LMA) is increasingly being used in children as it is less invasive compared to endotracheal intubation and causes less discomfort in the postoperative period. However, some concerns remained about its safety during positive pressure ventilation in children.

In a prospective randomized trial, 100 ASA I and II children weighing between 10-20 kg in the range of 2-10 years of age, scheduled for elective surgery were randomly allocated to one of the two groups of 50 patients each. The efficacy of LMA in children during positive pressure ventilation, its haemodynamic changes and postoperative complications were compared to endotracheal intubation. Insertion of LMA was easier in 94% patients while endotracheal intubation was done easily in 53% of patients only (p<0.05). The changes in haemodynamic parameters were significantly higher after endotracheal intubation as compared to LMA placement. Furthermore these changes persisted for longer duration after endotracheal intubation in comparison to LMA insertion (5 min vs 3 min). Incidence of postoperative complications i.e. bronchospasm, laryngospasm and soft tissue trauma was significantly higher (p<0.05) after endotracheal intubation as compared to LMA insertion.

To conclude, the laryngeal mask airway is a suitable alternative to endotracheal intubation for positive pressure ventilation in children.

## Introduction

The laryngeal mask airway (LMA), developed by Dr Archie I. J. Brain in 1983 provides a useful alternative for airway management during spontaneous or controlled ventilation in adults^1^. It is relatively non-invasive as compared to endotracheal intubation and causes minimal disturbances in cardiovascular and respiratory system^2^. Inflated cuff of LMA provides a low pressure seal over glottic opening and allow positive pressure ventilation (PPV) with inspiratory pressure of 15-20cm of water.

Recently there is increasing use of LMA in children because of ease of insertion and removal as compared to endotracheal intubation^3^,^4^ with minimal disturbances in cardiovascular and respiratory system and lesser risk of airway injury during the perioperative period^5^. However serious concerns remained about its efficacy in airway protection during positive pressure ventilation in children. In this study, we investigate the efficacy of LMA for positive pressure ventilation with size 2 LMA in children and compared it with endotracheal intubation.

## Methods

After taking permission from hospital ethics committee and with parental consent, we studied 100 ASA I & II patients of either sex weighing between 10-20 kilograms with age ranging between 2-10 years, scheduled for various elective surgical procedures of not more than 2 hours duration. The surgical procedures included in the study were herniotomy, cyst excision, suprapubic cystolithotomy, repair of hypospadias and urethroplasty. Exclusion criteria included a history of or anticipated difficult intubation, patient with pharyngeal pathology, patients with known pulmonary or cardiovascular disease. All patients were randomly allocated to one of the two groups of 50 patients each, applying computer generated randomization schedule. In Group A (ETT group) laryngoscopy and endotracheal intubation with appropriate sized uncuffed endotracheal tube (Romsons, India) was done while in Group B (LMA group) LMA-classic (Intavent, Berkshire) size 2 was inserted by the same anaesthetist in all patients. The person who performed these manoeuvres had an experience of more than 2 years duration in endotracheal intubation/ LMA placement. The cuff of LMA was inflated with less than 10 ml of air.

All patients were premedicated with ketamine 5mg.kg^−1^ and atropine 0.01 mg.kg^−1^ im followed by metoclopramide 0.2mg.kg^−1^ and midazolam 0.05mg.kg^−1^ iv. Induction of anaesthesia was done with an additional dose of ketamine as intravenous injection until unresponsive to pain followed by vecuronium bromide 0.08 mg.kg^−1^ iv to facilitate insertion of endotracheal tube/LMA. The position of endotracheal tube (ETT) or LMA was checked by observing movements of chest wall and auscultation for breath sounds during controlled ventilation. The efficacy of positive pressure ventilation was assessed by observing adequate chest rise on manual ventilation, bilateral equal air entry on auscultation and normal rectangular shape capnograph tracing. Ease of insertion of LMA/endotracheal tube was assessed as: Easy= successful at the first attempt; Difficult= successful but with some difficulty for any reason; Impossible= not successful. Number of attempts required for the proper placement of LMA/endotracheal tube was also recorded. Following successful ETT or LMA insertion, anaesthesia was maintained with N_2_O + O_2_ + halothane 0.5% and intermittent doses of vecuronium bromide as intravenous injections. Ventilation was controlled manually such as to maintain end tidal carbon di oxide concentration (EtCO_2_) between 35-45 mmHg.

Monitoring of airway pressure (kept below 15cm of water) and of vital signs i.e. heart rate, non-invasive blood pressure, pulse oximeter, EtCO_2_ and EKG lead-II via cardioscope was done during the perioperative period. Haemodynamic changes were recorded before induction (baseline), just after intubation (0 min), then at 1, 3 and 5 min after intubation. At the end of surgery the residual neuromuscular blockade was reversed with neostigmine 0.04mg.kg^−1^ and atropine 0.02 mg.kg^−1^ iv. Oxygenation was continued with a face mask in the recovery room. Patients were evaluated for change in the abdominal circumference just after the removal of ETT/LMA as compared to the preoperative circumference. Common complications like laryngospasm, bronchospasm, soft tissue trauma and aspiration during perioperative period were recorded by a blinded observer 24 hours after the completion of surgery.

For statistical analysis of data within the groups, paired student ‘t’ test was used while for comparison between groups' unpaired ‘t’ test was used. Results were considered statistically significant for p values <0.05. Postoperative complications were evaluated by using chi square test.

The sample size required for having a power of 80% for fulfilling primary goal (i.e. ease of insertion and efficacy of ventilation) of study was 46, based on previous studies. We had taken 50 patients in each group to obtain the power >80%.

## Results

The patients' demographic data were comparable in both groups (Table 1). The mean duration of surgery was also nearly identical in the two groups. Males were dominated in both the groups.

**Table 1:** Demographic Data

	Group A	Group B
Age in years (mean±SD)	5.8 ± 2.9	6.0 ± 3.0
Weight in kg (mean±SD)	15.7±4.7	16.2± 4.4
Duration of surgery in hours (mean±SD)	0.78± 0.33	0.72± 0.38
Sex ratio (M:F)	37:13	39:11

The LMA was placed in all patients in Group B. It was graded easy in 94% of patients and difficult in 6% of cases (Table 2). Endotracheal intubation was done in all patients in Group A; it was easy in 53% of patients and difficult in 47% of cases (Table 2). Number of attempts for correct placement of LMA was also significantly less as compared to endotracheal intubation (Table 3).

**Table 2:** Conditions during ETT/LMA insertion

	Group A	Group B	p value
Easy(E)	53%	94%	<0.05
Difficult(D)	47%	6%	<0.05
Impossible(I)	0%	0%	-

**Table 3:** Number of attempts for correct placement of ETT /LMA

No. of attempts	Group A	Group B	p value
1	53%	94%	<0.05
2	42%	4%	<0.05
>2	5%	2%	>0.05

Mean abdominal circumference increased significantly in the postoperative period (just after the removal of ETT/LMA) compared to the preoperative circumference in both groups of patients (p<0.05) as shown in Table 4. However the percent change in abdominal circumference for the LMA group was not significantly greater than that observed in the ETT group (p>0.05). The heart rate increased significantly in both groups after insertion of ETT/ LMA (p<0.01) as shown in Table 5. This increase in heart rate persisted up to 5 min in Group A (ETT group), while it came to baseline within 3 min in Group B (LMA group). The mean changes in heart rate at 0, 1, 3 min were highly significant in Group A as compared to Group B (p<0.001, <0.001, <0.05 at 0, 1 and 3 min respectively). There was significant increase in mean arterial pressure in both groups just after insertion of ETT / LMA. Like heart rate, the mean arterial pressure came back to baseline value after 5 min in Group A while within 3 min in Group B. Changes in mean arterial pressure in Group A at 0, 1, 3 min were significant as compared to Group B (p<0.001, <0.01, <0.01) (Table 6). There were no significant differences in SpO_2_ at 0, 1, 3, 5 min interval within the group as well as between the study groups.

**Table 4:** Changes in abdominal circumference

Abdominal Circumference	Group A	Group B
Initial(cm)	44.95 ± 1.98	45.87 ± 1.49
Final(cm)	45.45 ± 1.99	46.46 ± 1.36
p value	<0.05	<0.05

**Table 5:** Changes in heart rate at various time intervals(Mean±SD)

Groups (n=50)	P/I	0 min	1 min	3 min	5 min
**Group A**	104.9±8.2	114.6±8.1	134.4±7.7	122.2±4.6	105.7±8.3
p*		<0.01	<0.001	<0.01	NS
**Group B**	107.8±9.6	117.9±9.4	112.2±9.4	107.2±9.3	107.3±9.3
p*		<0.01	<0.001	NS	NS
p**	NS	<0.001	<0.001	<0.05	NS

P/I-Pre induction,

p* -Statistical significance within the group,

p** -Statistical significance between the groups., NS-Not significant

**Table 6:** Changes in mean arterial pressure at various time intervals(Mean±SD).

Groups (n=50)	PII	0 min	1 min	3 min	5 min
**Group A**	84.4±7.2	102.9±6.8	99.2±6.2	98.9±4.8	84.4±6.7
p*		<0.001	<0.001	<0.001	NS
**Group B**	81.2± 7.2	88.9±7.3	86.2±7.3	81.1±7.3	80.6±7.1
p*		<0.001	<0.001	NS	NS
p**	NS	<0.001	<0.001	<0.001	NS

P/I-Pre induction,

p* -Statistical significance within the group,

p** -Statistical significance between the groups., NS-Not significant

After removal of ETT or LMA, incidences of laryngospasm and bronchospasm in Group A (ETT) were 2% and 4% respectively but it was none in Group B (LMA) (p<0.05). The incidence of soft tissue trauma was significantly higher in group A (ETT) as compared to group B (LMA) (p<0.05), however gastric distension was noted 12% patients in group A and 14% in group B (p>0.05) (Table 7).

**Table 7:** Distribution of patients according to postoperative complications(%)

Complications	Group A	Group B	p values
Laryngospasm	2%	Nil	<0.05
Bronchospasm	4%	Nil	<0.05
Soft tissue trauma	10%	2%	<0.05
Aspiration	Nil	Nil	-
Gastric Distension	12%	14%	>0.05

## Discussion

In 1989 Brain stated that even though positive pressure ventilation (PPV) was “easily achieved” with LMA in adults, available data were insufficient to recommend its use in children. Subsequent reports described varied experiences with PPV in infants and children.^6^–^9^

Various anaesthetic techniques by different anaesthetists were used without the use of muscle relaxants. However the use of muscle relaxants improved the ease of insertion. In none of the cases LMA insertion failed, while it was 92.6% successful in previous studies^6^,^10^.

In our study, LMA size 2 was chosen for positive pressure ventilation in all patients in LMA group, as size 2 has been found appropriate for insertion and ventilation of children weighing between 10-20 kg. LMA was placed in first attempt in 94% cases, 4% required second attempt and 2% required third attempt. Various workers reported successful LMA placement on first attempt varying between 67%-92%. In our study, positive pressure ventilation was effective in 98% of patients. Excessive gas leak prevented PPV in one patient but repositioning and adjusting cuff volume reduced gas leak and improved ventilation. We did not experience any difficulty in carrying out mechanical ventilation through the LMA and airway pressure was kept below 18 cm of water. Earlier reports described PPV with LMA in children with^7^ or without^8^,^9^,^11^ the benefit of muscle relaxants.

The haemodynamic response to LMA insertion is much less as no laryngoscopy is needed which causes minimal changes in pulse rate and blood pressure upon insertion. The stress response consequent to its insertion is minimal as compared to tracheal intubation. As shown in the Tables 5 and 6 the HR and MAP in Group A (ETT group) raised for longer time period in comparison to Group B (LMA group). Other workers also drew same conclusion^2^.

Abdominal circumference was measured at the beginning and conclusion of the surgery. Mean abdominal circumference increased significantly in both the groups, but this increase for the LMA group was not significantly greater than that observed in the ETT group (p>0.05)^11^. Postoperative complications were noted at the time of removal of ETT or LMA. Incidences of laryngospasm and bronchospasm were 2% & 4% respectively in Group A (ETT) but none in Group B. Soft tissue trauma in Group A was seen in 10% of cases while in 2% of cases in Group B (p< 0.05). Similar incidences of complications were reported by Frediani et al^12^ and Tait et al^13^. As LMA is a less invasive airway device, the postoperative complications were less as compared to ETT. Aspiration was not observed in any case of Group A or Group B.

In conclusion, we can say that during routine paediatric use, LMA provides a satisfactory airway for PPV. Haemodynamic response is less and is short lived with LMA as compared to endotracheal intubation. Incidence of postoperative complications is also less with LMA than with ETT. Therefore LMA is a suitable alternative to endotracheal intubation for elective surgical procedures in paediatric patients.

## References

[1] Brain AIJ, McGhee TO, McAteer EJ (1985). The LMA. Anaesthesia.

[2] Wilson IG, Fell D, Robinson SL, Smith G (1992). Cardio vascular responses to insertion of the LMA. Anaesthesia.

[3] Goudsouzion NG, Denman W, Clevaland R, Shorten G (1992). Radiologic localization of the LMA in children. Anesthesiology.

[4] Watcha MF, White PF, Tychsen L, Stevens JL (1992). Comparative effects of LMA and ET insertion on intraocular pressure in children. Anesth Analg.

[5] Power M, Kohli M, Usha C, Gupta R (2001). A comparative study of the effects of placement of LMA vs ETT on haemodynamic parameters and IOP in children. Ind J Anaesth.

[6] Mason DG, Bingham RM (1990). Forum: the LMA in children. Anaesthesia.

[7] Asai J, Fujise K, Uchida M (1991). Use of LMA in a child with tracheal stenosis. Anesthesiology.

[8] Efrat R, Kadari A, Katsz S (1994). The LMA in paediatric anaesthesia: experience with 120 patients undergoing elective groin surgery. J Paediatr Surg.

[9] Burnett YL, Brennan MP, Salem MR (1994). Controlled ventilation and the LMA: Effect on end tidal CO_2_, O_2_ saturation, PIP and gastric distension in paediatric patients (abstract). Anesthesiology.

[10] Grebenik CR, Ferguson C, White A (1990). The LMA in paediatric radiotherapy. Anesthesiology.

[11] Gursoy F, Aigren JJ (1996). PPV with LMA in children. Anesth Analg.

[12] Frediani M, Blanchini G, Copanna M, Casini L, Costa M, Uggen S, Meinion, Pacini P (1996). The LMA in paediatric anaesthesia. Minerva Anesthesiol.

[13] Tait AR, Pandit UA, Voepet Lewis T, Munro HM, Malviya S (1998). Use of LMA in children with upper respiratory bacterial infections:a comparison with endotracheal intubation. Anesth Analg.

